# Dynapenic obesity and the effect on long-term physical function and quality of life: data from the osteoarthritis initiative

**DOI:** 10.1186/s12877-015-0118-9

**Published:** 2015-10-08

**Authors:** John A. Batsis, Alicia J. Zbehlik, Dawna Pidgeon, Stephen J. Bartels

**Affiliations:** Section of General Internal Medicine, Dartmouth-Hitchcock Medical Center, 1 Medical Center Drive, Lebanon, NH 03756 USA; Centers for Health and Aging, Dartmouth College, Lebanon, NH 03756 USA; Geisel School of Medicine at Dartmouth, Hanover, NH 03755 USA; Dartmouth Weight and Wellness Center, Lebanon, NH 03756 USA; Health Promotion Research Center at Dartmouth, Lebanon, NH 03756 USA; The Dartmouth Institute for Health Policy & Clinical Practice, Dartmouth College, Hanover, NH 03756 USA; Section of Rheumatology, Dartmouth-Hitchcock Medical Center, 1 Medical Center Drive, Lebanon, NH 03756 USA; Department of Rehabilitation, Lebanon, NH 03756 USA; Department of Community and Family Medicine, Lebanon, NH 03756 USA

**Keywords:** Dyanpenia, Obesity, Osteoarthritis, Disability, Muscle function

## Abstract

**Background:**

Obesity is associated with functional impairment, institutionalization, and increased mortality risk in elders. Dynapenia is defined as reduced muscle strength and is a known independent predictor of adverse events and disability. The synergy between dynapenia and obesity leads to worse outcomes than either independently. We identified the impact of dynapenic obesity in a cohort at risk for and with knee osteoarthritis on function.

**Methods:**

We identified adults aged ≥ 60 years from the Osteoarthritis Initiative. Obesity was defined as a body mass index ≥ 30 kg/m^2^. Dynapenia was classified using the lowest sex-specific tertile of knee extensor strength. Participants were grouped according to obesity and knee strength: dynapenic obesity; dynapenia without obesity; obesity without dynapenia; and no dynapenia nor obesity. Four-year data was available. Self-reported activities of daily living (ADL) were assessed at follow-up. Outcomes of gait speed, 400 m walk distance, Late-life Disability and Function Index (LLFDI), and Short-Form (SF)-12 were analyzed using mixed effects and logistic regression models.

**Results:**

Of 2025 subjects (56.3 % female), mean age was 68.2 years and 182 (24.1 %) had dynapenic obesity. Dynapenic obesity was associated with reduced gait speed, LLFDI-limitations, and SF-12 physical score in both sexes and in the 400 m walk in men only (all *p* < 0.001). A time*group interaction was significant for dynapenic obese men in the 400 m walk distance only. Odds of ADL limitations in dynapenic obesity was OR 2.23 [1.42:3.50], in dynapenia 0.98 [0.66:1.46], and in obesity 1.98 [1.39:2.80] in males. In females, odds were 2.45 [1.63:3.68], 1.60 [1.15:2.22], and 1.47 [1.06:2.04] respectively.

**Conclusion:**

Dynapenic obesity may be a risk factor for functional decline suggesting the need to target subjects with low knee strength and obesity.

**Electronic supplementary material:**

The online version of this article (doi:10.1186/s12877-015-0118-9) contains supplementary material, which is available to authorized users.

## Background

The obesity epidemic is observed even in an aging population with an overall prevalence in older United States adults of 35.4 % [1]. Both obesity and aging independently lead to adverse outcomes for older adults, including risk of long-term disability [2], institutionalization [3] and impaired quality of life [4]. Dynapenia, reflected by muscle weakness or low muscle strength, is a component of sarcopenia [5], a condition characterized by the loss of muscle mass during the aging process. Sarcopenia is associated with detrimental outcomes independent of aging and obesity [2]. Recent evidence suggests that mechanistic similarities underlie sarcopenia and obesity in aging [6]. The combination of these conditions in those surviving into old age creates additional challenges during the aging process, therefore, identifying people at high risk is extremely important in order to target specific interventions.

The synergy of sarcopenia and obesity leads to a high risk of adverse outcomes in affected individuals [7]. Challenges exist in adequately defining the relationship between sarcopenia and obesity which has impeded progress in characterizing the syndrome [5]. A number of studies have looked at the relationship between muscle mass, obesity and their long-term outcomes [2, 8, 9]. In contrast, dynapenia alone may also lead to adverse, unintended outcomes [10–13]. Previous studies have focused uniquely on cross-sectional relationships demonstrating the relationship of obesity and low muscle strength with impaired function [14, 15]. Emerging evidence has proven that dynapenic obesity (measured by waist circumference) may also be related to a higher risk of functional decline [14, 16] and death [17].

To our knowledge, there is very little data demonstrating the higher, yet theoretical, cumulative risk of dynapenia with obesity than with either disorder on its own. Understanding the natural history of patients with dynapenia and obesity is critically needed to allow clinicians to intervene in this at-risk population. Additionally, longitudinal data will allow a description of the time course of the observed decline, in particular, in a dataset that has a well characterized sample that includes both predictors and outcomes of interest. The purpose of this study was to characterize the effect of dynapenic obesity on physical functioning in a cohort at risk, and with osteoarthritis. We hypothesized that this subgroup is at risk for a faster decline in function and quality of life over time.

## Methods

We performed a secondary analysis of data using The Osteoarthritis Initiative (OAI), a multi-center, longitudinal, prospective observational study of people with knee osteoarthritis that begun in 2004. The study was funded through a public-private partnership whose goal was to evaluate the natural course and biomarkers of the onset and progression of knee osteoarthritis. There were four recruitment sites including Baltimore, MD, Columbus, OH, Pawtuckett, RI, and Pittsburgh, PA. Recruitment and enrollment procedures, which have been described elsewhere, were carried out within a 6-week time frame. Informed consent followed all pertinent federal guidelines with each component explained to potential participants, prior to screening or enrollment. Written consent was obtained prior to each clinic visit following thorough description of the study and its components by a trained staff member, answering questions, with a copy provided for review to participants before the scheduled visit. Documentation describing various aspects of the design and methods of the OAI is publically available on the OAI Online website (http://www.oai.ucsf.edu/). For this particular study, data at baseline, 1, 2, and 4 years were included. The local ethics committee (Institutional Review Board) at Dartmouth, the Committee for the Protection of Human Subjects, in Hanover, New Hampshire, exempted this present study from ethical approval due to the de-identified nature of the data. The OAI had a separate process approving the study, with each clinical center modifying the consent form template to comply with local Institutional requirements.

### Study cohort

**Fig. 1:**
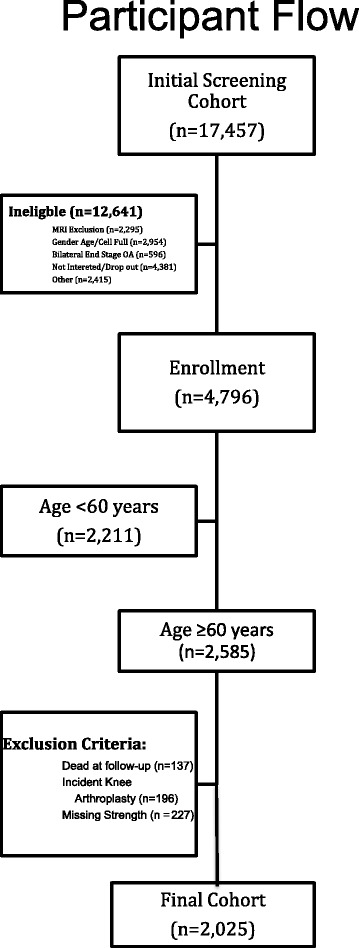
Participant Flow among 17,457 screened in the Osteoarthritis Initiative Protocol. Patient flow is demonstrated from initial telephone screen to cohort included in this study. Abbreviations: BMI—body mass index; MRI—magnetic resonance imaging; OA—osteoarthritis; WC—waist circumference

An ethnically diverse sample of men and women (age range 45–79 years) was interviewed for eligibility, and then assigned to a sub-cohort: subjects with clinically significant radiographic tibiofemoral knee OA in at least one native knee (progression cohort); subjects without symptomatic knee OA in either knee at baseline but consisting of established OA risk factors including Heberden nodes, previous knee operation or injury, family history, pain in the knee in the preceding month and weight defined using gender and age-specific cut-points (incidence cohort); and subjects without any pain or radiographic findings or risk factors (control cohort). OAI exclusion criteria consisted of individuals with rheumatoid arthritis, severe joint space narrowing, bilateral total knee arthroplasty, inability to undergo an MRI or able to provide a blood sample, co-morbidity interfering with study participation, individuals subject to moving from the study catchment area within 3 years, or other research participation. For this study, subjects <60 years of age were excluded (*n* = 2221) due to a lower risk of functional impairment [18] and higher capacity for homeostasis [19]. There were 560 subjects who died, had incident knee arthroplasty or had missing knee extensor strength values, and were therefore excluded as well. Participant flow is depicted in Fig. 1.

### Covariates

Standardized questionnaires, interviews, and assessments provided the basis for all self-reported variables and measurements. The age of the individual at the initial visit was considered baseline age. Education status was classified in four categories: attended high school with or without graduation, attended college, college graduate, or post-graduate education. Marital status was defined as single or married, where the former included subjects who lost a spouse, divorced, separated or never married. Ever smokers were defined as a person who smoked greater than 100 cigarettes in their lifetime. The Western Ontario and McMaster University OA Index (WOMAC) [20] assessed self-reported knee pain using a 5-point Likert scale about knee pain in each limb separately over the past 7 days, with scores ranging from 0 to 20. Higher scores represent worse symptoms. The Charlson co-morbidity index assessed subject co-morbidity [21]. All deaths were subject to a formal adjudication process through the OAI Coordinating Center.

Physical activity was defined using the Physical Activity Scale for the Elderly (PASE) scale [22], a 26-item instrument measuring occupational, household, and leisure activities during a 1-week period in older adults. This self-reported scale is reliable and valid, and can be administered by telephone, mail or in-person. Higher scores are associated with increased levels of activity and population-based means are available [22, 23]. Importantly, no minimally clinically important differences are available.

### Definition of dynapenic obesity

Weight was measured in kilograms using a calibrated standard balance beam scale. Subjects were asked to remove their shoes and heavy jewelry and wear light clothing. A wall-mounted stadiometer was used to measure standing height. Body mass index (BMI) was calculated as weight (in kilograms) divided by height (in meters) squared. Obesity was classified according to standard BMI categories: normal (18.5–24.9 kg/m^2^), overweight (25–29.9 kg/m^2^), and obese (≥30 kg/m^2^). Waist circumference (WC) was measured at the level of the umbilicus between the lower rib and the iliac crest. A high WC was classified as ≥88 cm or ≥102 cm in females and males, respectively [24]. Using a good strength chair with a supported back, knee extension was measured with the knee joint at a 60° angle measured by a goniometer. The transducer was centered behind the leg, with the bottom 2 cm above the calcaneous, placed behind the participant’s leg. After the leg was strapped, three trials at maximal effort were performed, measured in newtons (N). Full details are available online. Two practice trials were performed at 50 % effort, after a 15–20 min warm-up session. The greater of the left or right knee extensor strength was used for maximal knee strength. We classified dynapenia (yes/no) in each sex as participants in the lowest tertile of knee extensor strength (males: 365.8, 458.2 N; females 235.3; 304.1 N). Four categories were created based on these combinations.

### Self-reported outcomes

Perceived self-reported health was assessed using the SF-12, a shorter questionnaire accounting for >90 % of the statistical variance of the longer SF-36 [25]. A Likert scale assessed both physical (PCS) and mental (MCS) component scores. The score are standardized and weighted to a population mean of 50 ± 10. Higher scores represent better health.

Late-life Function and Disability Index (LLFDI) [26] is a self-reported instrument with two main domains, disability and function, each scored on a 0–100 scale, with higher scores indicating higher levels. The OAI measured the disability component, which is subdivided into the frequency subscale that describes an individual’s regularity of participation in life tasks, and a limitation scale that describes the capability to perform life tasks. Personal factors such as health, physical or mental energy, and environmental factors including transportation, accessibility and socio-economic conditions were probed. The instrument parallels the disablement framework described by Nagi on disability in community-dwelling older adults and correlates well with the physical functioning subscale of the Medical Outcomes Study 36-item Short Form (SF) Health Survey and the London Handicap Scale [27].

Activities of daily living (ADL) impairment was self-reported. Subjects were classified as having impairment in function if they were unable to perform a basic ADL including, walking, bathing, dressing, eating, transferring out of bed, or toileting [28].

### Objectively measured outcomes

Gait speed, a measure of functional performance that predicts impaired function and mortality [29], was assessed using the 20 m walk test at a usual walking pace. Subjects walked 20 m in an unobstructed dedicated corridor, turned around and walked in the opposite direction. This measure has excellent reliability and intra-class correlation. A minimally clinically significant difference of 0.1 m/s is considered clinically significant [30]. The 400 m walk test is a measure of aerobic capacity related to V0_2_ max that assesses physical fitness [31]. Approximately 98.1 % of participants that could complete the 20 m walk test were administered this measure. Heart rate and blood pressure were assessed, and questions related to recent cardiovascular history were posed to screen individuals. A similar course to that of the 20 m walk test was performed. Standard encouragement was given following every lap. As there were subjects who were unable to complete this test, the total distance traveled was used in lieu of the time to complete the test.

### Statistical analysis

All data was downloaded and merged into a single database in October 2014 for analysis. All continuous variables are represented as means ± standard deviations, and counts (percent). A one-way ANOVA assessed differences amongst baseline characteristics within each sex. Because functional measures and muscle strength are affected by sex, we elected to perform sex-specific analyses of all outcomes. A paired *t*-test assessed differences between baseline and follow-up at 4-years within each category (dynapenic obesity; dynapenia without obesity; obesity without dynapenia; Neither dynapenia nor obesity). An ANOVA was also used to compare mean scores between categories within a given time frame (either baseline or follow-up at 4-years). Within-person differences between baseline and follow-up were compared across the four categories to assess changes. Multiple comparison analyses were performed on all continuous variables in the unadjusted analyses (Bonferroni) with results comparing the dynapenic obesity group with all other subgroups (*df = 3*).

The primary outcome of interest was the association of the four categories of dynapenia and obesity over time with gait speed, 400 m walk, LLFDI and SF-12 subscales. Linear mixed models tested these associations on all four categories and time-main effects in addition to a time x dynapenia/obesity-group interaction. This method tested the differences between baseline and follow-up with changes over time. All models were adjusted for age, education, race, smoking status, PASE score, Charlson score, and cohort type (incidence, progression and control). We determined the impact of each dynapenia/obesity category on risk of incident mobility limitations by sex and by age group using logistic regression models. Odds ratios and 95 % confidence intervals were calculated. We classified those without dynapenia or obesity as the referent category. As an exploratory analysis, we stratified our analysis by age group (60–70years and 70+ years) to examine the impact of age. A sensitivity analysis compared subjects included vs. excluded. All data was analyzed using STATA version 12 (STATACorp, College Station, TX). A *p*-value <0.05 was considered statistically significant.

## Results

Baseline characteristics are presented in Table 1. Across all four categories, there were differences in age, socio-demographic factors, knee strength, BMI, and WOMAC scores. There were differences in females only in Charlson co-morbidity scores, smoking status, ADL impairments and cohort allocation. As compared to dynapenia alone, dynapenic obese patients had higher WOMAC scores with similar post-hoc comparisons in other baseline variables in both sexes. Additional file 1 outlines the differences between subjects included and excluded. Those excluded were older, had lower socioeconomic status, gait speed and SF-12 scores, and had gait speed and SF-12 scores, and had higher comorbid conditions and WOMAC scores. Table 2 displays the mean values at baseline and 48-month follow-up of our unadjusted primary outcome stratified by sex. Similar trends across categories were observed in both sexes. Significant changes were observed across groups in the 400 m walk (*p* < 0.001 and *p* = 0.03 in males and females, respectively), and differences in LLFDI-frequency and limitation subdomains. Dynapenic obese patients had a lower gait speed and SF-12 PCS score compared to those with dynapenia alone in both sexes at baseline. In males, baseline gait speed, follow-up 400 m walk test and SF-12 PCS score were significantly different in dynapenic obese subjects compared to those with dynapenia alone. In females, follow-up gait speed and SF-12 PCS scores were significantly different between dynapenic obese subjects compared to dynapenia alone. There were significantly higher unadjusted baseline 400 m walk and LLDFI-limitation scores at follow-up between individuals with obesity without dynapenia, as compared to dynapenic obese subjects in males alone. Faster (higher) follow-up gait speed and follow-up SF-12 PCS scores were observed in females with obesity without dynapenia as compared to females with dynapenic obesity.

**Table 1:** Baseline characteristics of the included cohort

			Males	*N* = 756				Females	*N* = 1269		
	Overall	Dynapenic obesity	Dynapenia no obesity	Obesity no dynapenia	Neither dynapenia nor obesity	*p*	Dynapenic obesity	Dynapenia no obesity	Obesity no dynapenia	Neither dynapenia nor obesity	*p*
	*N* = 2025	*N* = 68	*N* = 184	*N* = 186	*N* = 318		*N* = 114	*N* = 309	*N* = 296	*N* = 550	
Age, years	68.2 ± 5.4	69.4 ± 5.5	70.5 ± 4.9	67.4 ± 5.1^*^	68.7 ± 5.4	<0.001	67.5 ± 5.7	69.1 ± 5.7^*^	66.3 ± 4.9	67.8 ± 5.1	<0.001
Education status											
< High School	384 (18.9)	17 (25.0)	27 (14.8)	24 (13.0)	30 (9.4)		29 (25.4)	67 (21.7)	75 (25.5)	115 (21.0)	
Some College	483 (23.9)	11 (16.2)	23 (12.6)	41 (22.2)	44 (13.8)	0.001	41 (36.0)	74 (24.0)	100 (24.0)	149 (27.2)	0.01
College	395 (19.5)	19 (27.9)	37 (20.2)	44 (23.8)	79 (24.8)		16 (14.0)	63 (20.4)	46 (15.7)	91 (16.6)	
> College	756 (37.3)	21 (30.9)	96 (52.5)	76 (41.1)	165 (51.9)		28 (24.6)	105 (34.0)	73 (24.8)	192 (35.1)	
Yearly income											
> $50,000	1046 (51.7)	27 (43.6)	105 (59.0)	123 (68.0)	229 (74.4)	<0.001	36 (33.0)	146 (50.0)	111 (39.9)	269 (52.1)	<0.001
Marital status											
Married	1340 (66.2)	49 (72.1)	141 (77.1)	149 (80.5)	274 (86.2)	0.01	54 (47.4)	184 (59.6)	146 (49.8)	343 (62.7)	<0.001
Race											
White	1710 (84.4)	51 (75.0)	158 (85.9)	159 (85.5)	297 (93.4)		69 (60.5)	263 (85.1)	215 (72.6)	498 (90.6)	
Black	268 (13.2)	16 (23.5)	19 (10.3)	24 (12.9)	15 (4.7)	<0.001	43 (37.7)	40 (12.9)	72 (24.3)	39 (7.1)	<0.001
Other	47 (2.3	1 (1.5)	5 (2.7)	3 (1.6)	3 (0.9)		2 (1.8)	3 (1.0)	8 (2.7)	6 (1.1)	
Charlson score	0.43 ± 0.88	0.66 ± 1.22	0.60 ± 1.07	0.67 ± 1.12	0.45 ± 0.93	0.11	0.57 ± 0.93	0.31 ± 0.72^*^	0.53 ± 0.93	0.26 ± 0.60^#^	<0.001
PASE score	138.1 ± 67.3	123.5 ± 62.5	137.0 ± 68.9	150.3 ± 77.0^*^	160.3 ± 70.6^#^	<0.001	106.8 ± 64.0	122.9 ± 55.3	130.7 ± 63.9^*^	142.5 ± 65.4^#^	<0.001
Baseline WOMAC right	11.0 ± 13.6	14.1 ± 13.3	9.4 ± 10.2^*^	10.7 ± 13.4	6.9 ± 9.9^#^	<0.001	23.8 ± 21.1	13.2 ± 14.3^#^	14.4 ± 15.3^#^	7.9 ± 10.6^#^	<0.001
Baseline WOMAC left	10.6 ± 14.4	14.0 ± 15.0	8.8 ± 12.5^*^	11.3 ± 15.1	7.1 ± 11.2	<0.001	21.1 ± 20.2	11.4 ± 13.8^#^	14.1 ± 16.8^#^	8.1 ± 12.0^#^	<0.001
Ever smoker	999 (39.3)	40 (59.7)	105 (58.0)	97 (53.0)	168 (52.8)	0.54	41 (36.6)	126 (41.2)	153 (52.4)	269 (49.5)	0.003
No. medications	3.80 ± 2.46	5.0 ± 2.7	3.87 ± 2.51^*^	4.05 ± 2.74^*^	3.17 ± 2.08^#^	<0.001	4.47 ± 2.63	3.75 ± 2.62	4.21 ± 2.43	3.49 ± 2.23	<0.001
Body mass index, kg/m^2^	28.2 ± 4.5	33.1 ± 2.6	26.2 ± 2.3^#^	33.1 ± 2.8	26.6 ± 2.2^#^	<0.001	33.6 ± 3.2	24.9 ± 2.9^#^	33.5 ± 2.9	25.5 ± 2.8^#^	<0.001
Waist circumference, cm	103.1 ± 12.2	115.5 ± 8.8	99.3 ± 7.6^#^	115.3 ± 8.4	99.6 ± 7.3^#^	<0.001	113.5 ± 10.7	96.1 ± 10.1^#^	113.8 ± 10.1	96.9 ± 9.8^#^	<0.001
High waist circumference	1538 (76.0)	63 (95.5)	72 (39.3)	179 (96.8)	114 (36.4)	<0.001	114 (100.0)	243 (78.9)	295 (100.0)	458 (83.6)	<0.001
Knee extensor strength, N	326.4 ± 117.1	302.4 ± 53.4	302.0 ± 50.6	483.4 ± 90.4^#^	471.9 ± 82.7^#^	<0.001	185.1 ± 42.1	184.9 ± 38.5	321.7 ± 61.1^#^	311.7 ± 54.3^#^	<0.001
Cohort allocation											
Incidence	1492 (73.7)	43 (63.2)	142 (77.2)	124 (66.7)	242 (76.1)		56 (49.1)	236 (76.4)	197 (66.6)	450 (81.8)	
Progression	526 (26.0)	25 (36.8)	41 (22.3)	61 (32.8)	73 (23.0)	0.06	58 (50.9)	72 (23.3)	99 (33.5)	99 (18.0)	<0.001
Control	7 (0.4)	---	1 (0.5)	1 (0.5)	3 (0.9)		---	1 (0.2)	---	1 (0.2)	
ADL impairment	109 (7.2)	8 (16.7)	10 (20.8)	15 (31.3)	15 (31.3)	0.06	11 (18.0)	15 (24.6)	17 (27.9)	18 (29.5)	0.003

All values are represented as mean ± standard deviation, or count (%)

*P*-value represents the ANOVA across all body mass index categories

*p*-values represent analysis of variance between 4 quartile categories in each sex

Obesity represented as BMI ≥ 30 kg/m^2^; Dynapenia represented as lowest tertile in Males (Knee extensor strength < 365.8 N, and in Females (<235.3 N)

High waist circumference is ≥88 cm in females*;* ≥102 cm in males

Multiple comparison analyses performed between Dynapenic Obesity group and other groups: ^*^ *P* < 0.05; ^#^ *P* < 0.001

*ADL* activities of daily living, *PASE* physical activity for the elderly, *WOMAC* Western Ontario McMaster Universities Arthritis Index

**Table 2:** Sex-specific unadjusted functional outcomes by dynapenia/obesity category—baseline and 48-month follow-up

			Males	*N* = 756				Females	N = 1269		
		Dynapenic obesity	Dynapenia no obesity	Obesity no dynapenia	Neither dynapenia nor obesity	*p* ^b^	Dynapenic obesity	Dynapenia no obesity	Obesity no dynapenia	Neither dynapenia nor obesity	*p* ^b^
		*N* = 68	*N* = 184	*N* = 186	*N* = 318		*N* = 114	*N* = 309	*N* = 296	*N* = 550	
Gait Speed	Baseline	1.21 ± 0.15	1.30 ± 0.21^#^	1.28 ± 0.19^*^	1.39 ± 0.18^*^	<0.001	1.11 ± 0.23	1.27 ± 0.23^#^	1.22 ± 0.19^#^	1.33 ± 0.19^#^	<0.001
	Follow-up	1.19 ± 0.19	1.25 ± 0.20	1.26 ± 0.18	1.36 ± 0.19^#^	<0.001	1.08 ± 0.22	1.23 ± 0.22^#^	1.19 ± 0.19^#^	1.31 ± 0.18^#^	<0.001
	*p*-value^a^	0.05	0.009	0.05	<0.001	0.60^c^	0.03	<0.001	<0.001	<0.001	0.32^c^
400 M Walk	Baseline	399.4 ± 4.9	399.3 ± 8.9	398.6 ± 19.3	398.7 ± 17.1	0.95	388.0 ± 54.5	397.8 ± 24.4^*^	394.1 ± 38.7^#^	397.7 ± 22.5*	0.01
	Follow-up	366.1 ± 87.6	395.6 ± 30.4^#^	395.4 ± 29.4^#^	398.8 ± 18.0^#^	<0.001	383.6	395.7 ± 35.4	387.0 ± 51.0	394.0 ± 36.6	0.05
	*p*-value^a^	0.01	0.20	0.07	0.59	<0.001^c^	0.04	0.08	<0.001	0.003	0.03^c^
LLDFI-Frequency	Follow-up	52.6 ± 6.9	53.5 ± 6.3	53.9 ± 6.4	53.9 ± 5.1	0.004	54.5 ± 7.8	56.5 ± 6.3	55.1 ± 5.8	57.3 ± 6.5^*^	<0.001
LLDFI Limitations	Follow-up	75.3 ± 15.2	80.6 ± 15.2	82.4 ± 14.7^*^	85.2 ± 14.9^#^	<0.001	75.2 ± 17.3	79.5 ± 14.2	78.5 ± 15.3	82.9 ± 14.5^#^	<0.001
SF-12 PCS	Baseline	43.8 ± 9.6	49.7 ± 7.6^#^	48.6 ± 7.9^#^	51.5 ± 7.0^#^	<0.001	42.0 ± 10.9	48.2 ± 9.0^#^	47.6 ± 9.0^#^	50.8 ± 7.9^#^	<0.001
	Follow-up	41.0 ± 11.2	47.1 ± 8.5^#^	46.8 ± 8.7	^#^49.9 ± 8.0^#^	<0.001	40.7 ± 11.5	47.1 ± 9.0^*^	45.3 ± 10.0^#^	49.5 ± 8.3^#^	<0.001
	*p*-value^a^	0.003	<0.001	<0.001	<0.001	0.38^c^	0.09	0.001	<0.001	<0.001	0.20^c^
SF-12 MCS	Baseline	55.1 ± 9.6	55.2 ± 7.6	55.2 ± 7.9	56.2 ± 7.0	0.006	53.3 ± 10.9	54.7 ± 9.0	53.7 ± 9.0	54.6 ± 7.9	0.17
	Follow-up	55.4 ± 7.2	55.3 ± 8.0	54.5 ± 8.5	55.5 ± 6.9	0.59	54.6 ± 10.3	54.1 ± 8.1	53.5 ± 9.4	54.3 ± 7.6	0.59
	*p*-value^a^	0.52	0.89	0.25	0.13	0.50^c^	0.52	0.06	0.82	0.14	0.42^c^

Physical and Mental Component Scores are part of the Short-Form 12 assessment

Obesity represented as BMI ≥ 30 kg/m^2^; Dynapenia represented as lowest tertile in Males (Knee extensor strength <365.8 N, and in Females (<235.3 N)

A decrease in Gait Speed and 400 m walk test, represent reductions in mobility speed and fitness. Higher scores of Late-life function and disability scores represent better function (or less disability). A drop in Short-Form 12 score (physical and mental) represents a reduction in self-reported health status

Multiple comparison analyses performed between Dynapenic Obesity group and other groups: ^*^ *P* < 0.05; ^#^ *P* < 0.001

*LLFDI* late-life function & disability index, *MCS* mental component, *PCS* physical component, *SF* short form

All values represented are means ± standard deviation or count (%)

^a^ *p*-values within groups represent significance of change from baseline to follow-up

^b^ *p*-values represent overall test of difference in means between groups

^c^ *P*-values represent differences in change from baseline to follow-up between groups

Tables 3 and 4 demonstrate the sex-specific linear mixed effects modeling analysis. In both males and females, there were significant reductions in the dynapenic obesity group, as compared to those with neither dynapenia nor obesity, in gait speed, LLFDI-limitations subscale and SF-12 PCS scores. In males only with dynapenic obesity, reductions were observed in the 400 m walk test and LLFDI components only. We observed a significant time x dynapenia/obesity interaction in males for the 400 m walk test but not in any other measures or in the female sex. This finding highlights the rate of the decline in this measure alone in males only. No measures reached statistical significance in any of the interaction terms in females. Additional file 2a and b represent adjusted age-stratified functional outcomes by sex. In both age strata (60–70years, ≥70 years), both males and females with dynapenic obesity, as compared to those without dynapenia nor obesity, had lower gait speeds and SF-12 PCS scores. Subjects aged ≥70 years had lower gait speeds in both sexes, but significant differences in LLDFI and 400 m walk components in males alone. Decline over time (time x dynapenia/obesity interaction) was observed in both sexes in the ≥70 year age group. We did not observe any differences in the MCS score. We detected increased risk of mobility limitations in both males and females for those with dynapenic obesity at baseline, as compared to those without dynapenia nor obesity (Table 5). The relationship was more pronounced in those aged 70 years and older.

**Table 3:** Multivariable regression analysis of primary outcome measures (*n* = 756)—males

	Gait speed			400 m walk			LLFDI-Frequency			LLFDI-Limitation			SF-12 PCS			SF-12 MCS		
	β	SE	*p*	β	SE	*p*	β	SE	*p*	β	SE	*p*	β	SE	*p*	β	SE	*p*
Intercept	1.77	0.10	<0.001	411.5	12.33	<0.001	53.52	1.73	<0.001	109.4	4.61	<0.001	52.6	3.9	<0.001	54.1	3.49	<0.001
Age	−0.008	0.001	<0.001	−0.438	0.147	0.003	−0.103	0.021	<0.001	−0.481	0.056	<0.001	−0.162	0.05	0.001	0.031	0.041	0.46
Dynapenic obesity	−0.137	0.023	<0.001	−10.3	2.80	<0.001	1.55	0.419	<0.001	−5.00	1.12	<0.001	−6.12	0.89	<0.001	0.186	0.79	0.81
Dynapenia no obesity	−0.048	0.016	0.002	1.57	1.90	0.41	−0.63	0.26	0.15	−3.14	0.69	<0.001	−1.15	0.61	0.06	−0.101	0.54	0.85
Obesity no dynapenia	−0.087	0.016	<0.001	−0.97	1.90	0.61	−0.27	0.26	0.29	−4.44	0.69	<0.001	−2.40	0.61	<0.001	−0.931	0.54	0.09
Neither dynapenia nor obesity	Ref	Ref	Ref	Ref	Ref	Ref	Ref	Ref	Ref	Ref	Ref	Ref	Ref	Ref	Ref	Ref	Ref	Ref
Time	−0.01	0.002	<0.001	−0.141	0.430	0.74	--	--	--	--	--	--	−0.44	0.10	<0.001	−0.097	0.09	0.29
x Dyn obes	−0.0012	0.004	0.78	−8.07	1.07	<0.001	--	--	--	--	--	--	−0.05	0.24	0.84	0.033	0.22	0.88
X Dyn no obes	−0.008	0.003	0.79	−0.775	0.71	0.28	--	--	--	--	--	--	−0.35	0.16	0.03	0.165	0.15	0.27
x Obes no dyn	0.0026	0.003	0.37	−0.79	0.716	0.27	--	--	--	--	--	--	−0.06	0.16	0.70	−0.077	0.15	0.61
x No dyn no obes	Ref	Ref	Ref	Ref	Ref	Ref	--	--	--	--	--	--	Ref	Ref	Ref	Ref	Ref	Ref

All linear mixed models are adjusted for age, physical activity (Physical Activity Scale for the Elderly Score), smoking status, Charlson co-morbidity score, education, race, cohort type (incidence, progression, control). Referent category is the Neither dynapenia nor obesity group. Time-dependent co-variates are included in time x group interaction. LLDI was only available at 4-year follow-up thereby no time interaction term model was considered for this measure

*Dyn* dynapenia, *Obes* obesity, *β* beta-coefficient of regression model, *SE* standard errors, *LLDI* late-life functional and disability index, *MCS* mental component score, *PCS* physical component score, *SF* short form

**Table 4:** Multivariable regression analysis of primary outcome measures (*n* = 1269)—females

	Gait speed			400 m walk			LLFDI-frequency			LLFDI-Limitation			SF-12 PCS			SF-12 MCS		
	β	SE	*p*	β	SE	*p*	β	SE	*p*	β	SE	*p*	β	SE	*p*	β	SE	*p*
Intercept	1.748	0.080	<0.001	454.9	15.5	<0.001	56.3	1.49	<0.001	85.1	3.48	<0.001	51.5	3.47	<0.001	44.2	3.29	<0.001
Age	−0.009	0.001	<0.001	−0.90	0.18	<0.001	−0.044	0.168	0.009	−0.267	0.039	<0.001	−0.13	0.04	0.001	0.067	0.04	0.08
Dynapenic obesity	−0.141	0.018	<0.001	−6.34	3.61	0.08	−0.685	0.345	0.05	−4.34	0.808	<0.001	−5.15	0.79	<0.001	−0.612	0.75	0.42
Dynapenia no obesity	−0.040	0.012	0.001	1.66	2.33	0.48	−0.263	0.22	0.24	−2.38	0.52	<0.001	−1.90	0.52	<0.001	−0.590	0.50	0.24
Obesity no dynapenia	−0.082	0.013	<0.001	−4.17	2.43	0.09	−0.743	0.23	0.001	−3.67	0.54	<0.001	−2.42	0.55	<0.001	−0.275	0.52	0.60
Neither dynapenia nor obesity	Ref	Ref	Ref	Ref	Ref	Ref	Ref	Ref	Ref	Ref	Ref	Ref	Ref	Ref	Ref	Ref	Ref	Ref
Time	−0.0083	0.001	<0.001	−1.23	0.46	0.007	---	---	---	---	---	---	−0.38	0.08	<0.001	−0.109	0.077	0.15
x Dyn obes	−0.0042	0.004	0.24	−2.25	1.22	0.07	---	---	---	---	---	---	0.07	0.19	0.71	0.234	0.19	0.22
X Dyn no obes	−0.0047	0.0024	0.05	0.063	0.773	0.94	---	---	---	---	---	---	−0.05	0.13	0.70	−0.086	0.13	0.51
x Obes no dyn	0.0005	0.0024	0.85	−1.40	0.78	0.07	---	---	---	--	---	---	−0.32	0.13	0.01	0.126	0.13	0.13
x No dyn no obes	ref	ref	ref	Ref	Ref	ref	---	---	---	---	---	---	Ref	Ref	Ref	Ref	Ref	Ref

All linear mixed models are adjusted for age, physical activity (Physical Activity Scale for the Elderly Score), smoking status, Charlson co-morbidity score, education, race, cohort type (incidence, progression, control). Referent category is the neither dynapenia nor obesity group. Time-dependent co-variates are included in time x group interaction. LLDI was only available at 4-year follow-up thereby no time interaction term model was considered for this outcome measure

*Dyn* dynapenia, *Obes* Obesity, *β* beta-coefficient of regression model, *SE* standard errors, *LLDI* late-life functional and disability index, *MCS* mental component score, *PCS* physical component score, *SF* short form

**Table 5:** Odds ratio of incident mobility limitation by category

		Dynapenic obesity	Dynapenia no obesity	Obesity no dynapenia	Neither dynapenia nor obesity
Males	Unadjusted	3.12 [2.07:4.71]	1.26 [0.87:1.82]	1.92 [1.38:2.68]	Ref
	Adjusted	2.23 [1.42:3.50]	0.98 [0.66:1.46]	1.98 [1.39:2.80]	Ref
Females	Unadjusted	4.26 [2.98:6.08]	1.73 [1.26:2.37]	1.83 [1.34:2.48]	Ref
	Adjusted	2.45 [1.63:3.68]	1.60 [1.15:2.22]	1.47 [1.06:2.04]	Ref
60–70 years	Unadjusted	3.07 [2.07:4.56]	1.31 [0.89:1.92]	1.95 [1.44:2.62]	Ref
	Adjusted	1.92 [1.23:3.01]	1.12 [0.74:1.70]	1.65 [1.20:2.27]	Ref
70+ years	Unadjusted	4.43 [3.05:6.45]	1.49 [1.09:2.05]	1.94 [1.36:2.74]	Ref
	Adjusted	3.06 [2.01:4.66]	1.36 [0.98:1.88]	1.96 [1.36:2.83]	Ref

All models are adjusted for physical activity (Physical Activity Scale for the Elderly Score), smoking status, Charlson co-morbidity score, education, race, cohort type (incidence, progression, control)

For sex-specific models, age is an additional co-variate; for age-specific models, sex is an additional co-variate

Referent category is the neither dynapenia nor obesity group

## Discussion

Our study provides added longitudinal evidence that dynapenic obesity, as defined by reduced knee extensor strength and a BMI ≥ 30 kg/m^2^, leads to reduced physical function, higher disability, and lower quality of life in older adults at risk for and with osteoarthritis of the knee. Additionally, this subgroup is strongly associated with increased risk of ADL impairment in both sexes over time.

Previous cross-sectional studies demonstrate a disparate interplay between sarcopenia, dynapenia, obesity and the impact on important geriatric measures in late-life [14, 16, 17, 32–37]. These results add to the emerging data that may convince policy makers of the true impact of this epidemic. For instance, if older adults with dynapenic obesity have a 2–3 fold higher risk of ADL limitations, this adversely impacts independence and can lead to death or institutionalization [38, 39]. Understanding the natural course of the disease will inform practitioners and researchers as they develop interventions specifically targeted at the affected population.

Not surprisingly, the majority of our outcomes dropped from baseline to 48-month follow-up. Gait speed dropped over time in both sexes but more so in the dynapenic obesity group. This is consistent with other studies demonstrating the drop in gait speed and physical fitness with time in the overall population [30]. What was striking were the changes in SF-12 physical functioning and LLFDI-limitation subscales in both sexes, corresponding to the association of quality of life with physical functioning measures. Yet, our results show that MCS is not impacted by dynapenic obesity in this population.

Introducing a time x dynapenia/obesity group interaction term in this cross-sectional time series analysis allowed us to benefit from a number of advantages of linear mixed-effects regression models including: a) the ability to model individual change across time; b) the ability to incorporate measures without complete data that is missing at random, making it a superior statistical measure over a repeated-measures ANOVA (ie: not requiring complete case ascertainment); and c) the capacity to incorporate variables at different time periods. While differences were observed between categories, we were surprised that despite a considerable number of primary outcomes, including gait speed, 400 m walk, and SF-12, only the 400 m walk distance significantly declined in those with dynapenic obesity. These suggest three possible phenomena. First, the divergent scores in each measure likely occurred before 60 years of age since these differences were present at baseline. Second, the rate of change for the 400 m walk test differed significantly in those with dynapenia in those aged ≥70 years implying that a decline is observed later in life as well. Third, this cohort excluded those with TKA, death, low BMI and were community-dwelling adults participating in a research study. A number of subjects were excluded who had considerable co-morbidity, lower socioeconomic status, and pain. A comparison analysis of missing vs. included subjects was performed and confirmed that our cohort may have been healthier and thus we may not have observed the magnitude of the expected trends. The trajectories parallel other population groups, including one with a normal BMI with central adiposity, where the magnitudes of such declines occurred earlier in life [40]. Observing these differences in larger and older populations can better define some of these trajectories.

The Foundation for the National Institutes for Health Sarcopenia consensus suggest the use of two measures to account for sarcopenia: appendicular skeletal mass and grip strength [5]. The former can only be measured using sophisticated research tools and clinically is impractical; the latter can easily be incorporated into a busy primary care practice using a dynamometer as a component of sarcopenia. A number of studies have demonstrated the relationship between knee extensor strength [16, 41–43] and grip strength [44, 45] on adverse outcomes in older adults. Knee extensor strength is often available in research centers and training facilities, and has been proven in one study to be superior to that of grip strength in assessing strength in assisted living populations [46]. Future study in this population should examine grip strength, in lieu of knee extensor strength, as a marker for dynapenia.

Classification bias is an overarching concern in a number of studies examining prevalence and outcomes of dynapenic obesity [47]. Our study is no exception. First, we used knee extensor strength and classified low strength as subjects in the lowest sex-specific tertile. While other authors have used similar approaches [16], ideally, national, population-specific norms of low knee extensor strength are needed and should be used. Secondly, we fully acknowledge that we used BMI as a measure of obesity and that this anthropometric measure, while easy to use clinically, may suboptimally assess fat in older adults [48]. We considered using waist circumference, however, the majority of participants (>75 %) had an elevated waist circumference based on criteria. Third, ideally a comparison of dynapenic obesity defined using either low grip strength or low appendicular skeletal muscle mass adjusted by BMI as proposed by Studenski should be considered in future study designs [5]. Fourth, normalization of knee extensor strength by muscle mass could account for differences in strength based on body size. Without full body composition data, this approach would not allow us to best understand the impact of dynapenic obesity on our outcome measures. Lastly, changes in body composition with aging are known to impact future risk of future function and disability which could not be accounted in this particular analysis.

The dataset was initially meant to observe the differences between three distinct subgroups on risk factors and progression of osteoarthritis. By stratifying our results into four categories by sex, we lost considerable statistical power in our modeling to be able to compare the effect of dynapenic obesity on the three subgroups. Inherently, the information obtained from such a study would be very important and critical in the understanding of the natural history, progression and possible mechanisms to incident disability and their trajectory observed in clinical practice. Future studies with adequate sample size can identify the absolute changes and rates of declines in those with and without knee OA.

We recognize that this study has a number of other limitations. Clinical studies risk participants dropping out, and often dropouts have higher degrees of co-morbidity and lower socioeconomic status potentially biasing our results. A 4-year time period may not be sufficient to observe the impact of these results in a relatively ‘young’ population with minimal co-morbidity as reflected by the Charlson co-morbidity index. In fact, our age-stratified analysis suggested that age indeed is a factor on these important geriatric outcomes. Other parameters that clearly influence quality of life and objective measurable outcomes, including the degree of depression, involvement of hip osteoarthritis, and muscle circumference could be incorporated in future analysis with increased study power. While these could be incorporated in the current study, we would run the risk of over-adjustment. Lastly, we relied on self-reported and non-standardized functional assessments (ADLs) which may impact our estimates. Future study should consider identifying biomarkers associated with both dynapenia and obesity that could possibly explain the mechanisms involved in this trajectory.

## Conclusion

Dynapenia with obesity is associated with adverse objective and self-reported functional outcomes and reduced physical functioning and self-reported health. Encouraging such patients to engage in tailored interventions consisting of caloric restriction, regular resistance training, and targeted nutritional supplementation, should be considered to improve overall performance and reduce the risk of disability.

## Additional files

Additional file 1:
**Sensitivity analysis of excluded vs. Included participants.** (DOCX 15 kb) Additional file 2:
**a: Age-Stratified Functional Outcome - Males.** b: Age-Stratified Functional Outcome - Females. (DOCX 34 kb)

## Abbreviations

ADL: Activities of daily living
BMI: Body mass index
LLDFI: Late-life disability and function index
MCS: Mental component scale
OA: Osteoarthritis
OAI: Osteoarthritis initiative
PASE: Physical activity scale for the elderly
PCS: Physical component scale
SF: Short form
WC: Waist circumference
WOMAC: Western Ontario and McMaster University Osteoarthritis Index

## References

[1] Ogden CL, Carroll MD, Kit BK, Flegal KM (2014). Prevalence of childhood and adult obesity in the United States, 2011-2012. JAMA.

[2] Schaap LA, Koster A, Visser M (2012). Adiposity, muscle mass, and muscle strength in relation to functional decline in older persons. Epidemiol Rev.

[3] Zizza CA, Herring A, Stevens J, Popkin BM (2002). Obesity affects nursing-care facility admission among whites but not blacks. Obes Res.

[4] Rosemann T, Grol R, Herman K, Wensing M, Szecsenyi J (2008). Association between obesity, quality of life, physical activity and health service utilization in primary care patients with osteoarthritis. Int J Behav Nutr Phys Act.

[5] Studenski SA, Peters KW, Alley DE, Cawthon PM, McLean RR, Harris TB, Ferrucci L, Guralnik JM, Fragala MS, Kenny AM (2014). The FNIH sarcopenia project: rationale, study description, conference recommendations, and final estimates. J Gerontol A Biol Sci Med Sci.

[6] Stenholm S, Harris TB, Rantanen T, Visser M, Kritchevsky SB, Ferrucci L (2008). Sarcopenic obesity: definition, cause and consequences. Cur Opin Clin Nutr Metab Care.

[7] Cruz-Jentoft AJ, Baeyens JP, Bauer JM, Boirie Y, Cederholm T, Landi F, Martin FC, Michel JP, Rolland Y, Schneider SM (2010). Sarcopenia: European consensus on definition and diagnosis. Age Ageing.

[8] Kim TN, Park MS, Ryu JY, Choi HY, Hong HC, Yoo HJ, Kang HJ, Song W, Park SW, Baik SH (2014). Impact of visceral fat on skeletal muscle mass and vice versa in a prospective cohort study: the Korean Sarcopenic Obesity Study (KSOS). PLoS One.

[9] Lim S, Kim JH, Yoon JW, Kang SM, Choi SH, Park YJ, Kim KW, Lim JY, Park KS, Jang HC (2010). Sarcopenic obesity: prevalence and association with metabolic syndrome in the Korean Longitudinal Study on Health and Aging (KLoSHA). Diabetes Care.

[10] Clark BC, Manini TM (2012). What is dynapenia?. Nutrition.

[11] Manini TM, Clark BC (2012). Dynapenia and aging: an update. J Gerontol A Biol Sci Med Sci.

[12] Metter EJ, Talbot LA, Schrager M, Conwit R (2002). Skeletal muscle strength as a predictor of all-cause mortality in healthy men. J Gerontol A Biol Sci Med Sci.

[13] Newman AB, Kupelian V, Visser M, Simonsick EM, Goodpaster BH, Kritchevsky SB, Tylavsky FA, Rubin SM, Harris TB (2006). Strength, but not muscle mass, is associated with mortality in the health, aging and body composition study cohort. J Gerontol A Biol Sci Med Sci.

[14] Scott D, Sanders KM, Aitken D, Hayes A, Ebeling PR, Jones G (2014). Sarcopenic obesity and dynapenic obesity: 5-year associations with falls risk in middle-aged and older adults. Obesity (Silver Spring).

[15] Yang M, Jiang J, Hao Q, Luo L, Dong B (2015). Dynapenic obesity and lower extremity function in elderly adults. J Am Med Dir Assoc.

[16] Stenholm S, Alley D, Bandinelli S, Griswold ME, Koskinen S, Rantanen T, Guralnik JM, Ferrucci L (2009). The effect of obesity combined with low muscle strength on decline in mobility in older persons: results from the InCHIANTI study. Int J Obes (Lond).

[17] Rossi AP, Fantin F, Caliari C, Zoico E, Mazzali G, Zanardo M, Bertassello P, Zanandrea V, Micciolo R, Zamboni M. Dynapenic abdominal obesity as predictor of mortality and disability worsening in older adults: a 10-year prospective study. Clin Nutr 2015. doi: 10.1016/j.clnu.2015.02.005. 10.1016/j.clnu.2015.02.005 25736030

[18] Dunlop DD, Hughes SL, Manheim LM (1997). Disability in activities of daily living: patterns of change and a hierarchy of disability. Am J Public Health.

[19] Fried LP, Tangen CM, Walston J, Newman AB, Hirsch C, Gottdiener J, Seeman T, Tracy R, Kop WJ, Burke G (2001). Frailty in older adults: evidence for a phenotype. J Gerontol A Biol Sci Med Sci.

[20] Baron G, Tubach F, Ravaud P, Logeart I, Dougados M (2007). Validation of a short form of the Western Ontario and McMaster Universities Osteoarthritis Index function subscale in hip and knee osteoarthritis. Arthritis Rheum.

[21] Charlson ME, Pompei P, Ales KL, MacKenzie CR (1987). A new method of classifying prognostic comorbidity in longitudinal studies: development and validation. J Chronic Dis.

[22] Washburn RA, Ficker JL (1999). Physical Activity Scale for the Elderly (PASE): the relationship with activity measured by a portable accelerometer. J Sports Med Phys Fitness.

[23] Svege I, Kolle E, Risberg MA (2012). Reliability and validity of the Physical Activity Scale for the Elderly (PASE) in patients with hip osteoarthritis. BMC Musculoskelet Disord.

[24] Batsis JA, Nieto-Martinez RE, Lopez-Jimenez F (2007). Metabolic syndrome: from global epidemiology to individualized medicine. Clin Pharmacol Ther.

[25] Ware J, Kosinski M, Keller S (1995). How to score the SF-12 physical. The mental health summary scales.

[26] Sayers SP, Jette AM, Haley SM, Heeren TC, Guralnik JM, Fielding RA (2004). Validation of the late-life function and disability instrument. J Am Geriatr Soc.

[27] Harwood RH, Rogers A, Dickinson E, Ebrahim S (1994). Measuring handicap: the London Handicap Scale, a new outcome measure for chronic disease. Qual Health Care.

[28] Katz S, Ford AB, Moskowitz RW, Jackson BA, Jaffe MW (1963). Studies of illness in the aged. The index of ADL: a standardized measure of biological and psychosocial function. JAMA.

[29] Lopopolo RB, Greco M, Sullivan D, Craik RL, Mangione KK (2006). Effect of therapeutic exercise on gait speed in community-dwelling elderly people: a meta-analysis. Phys Ther.

[30] Studenski S, Perera S, Patel K, Rosano C, Faulkner K, Inzitari M, Brach J, Chandler J, Cawthon P, Connor EB (2011). Gait speed and survival in older adults. JAMA.

[31] Rolland YM, Cesari M, Miller ME, Penninx BW, Atkinson HH, Pahor M (2004). Reliability of the 400-m usual-pace walk test as an assessment of mobility limitation in older adults. J Am Geriatr Soc.

[32] Aubertin-Leheudre M, Lord C, Goulet ED, Khalil A, Dionne IJ (2006). Effect of sarcopenia on cardiovascular disease risk factors in obese postmenopausal women. Obesity (Silver Spring).

[33] Baumgartner RN, Wayne SJ, Waters DL, Janssen I, Gallagher D, Morley JE (2004). Sarcopenic obesity predicts instrumental activities of daily living disability in the elderly. Obes Res.

[34] Bouchard DR, Dionne IJ, Brochu M (2009). Sarcopenic/obesity and physical capacity in older men and women: data from the Nutrition as a Determinant of Successful Aging (NuAge)-the Quebec longitudinal study. Obesity.

[35] Janssen I, Mark AE (2006). Separate and combined influence of body mass index and waist circumference on arthritis and knee osteoarthritis. Int J Obes (Lond).

[36] Newman AB, Kupelian V, Visser M, Simonsick E, Goodpaster B, Nevitt M, Kritchevsky SB, Tylavsky FA, Rubin SM, Harris TB (2003). Sarcopenia: alternative definitions and associations with lower extremity function. J Am Geriatr Soc.

[37] Rolland Y, Lauwers-Cances V, Cristini C, Abellan van Kan G, Janssen I, Morley JE, Vellas B (2009). Difficulties with physical function associated with obesity, sarcopenia, and sarcopenic-obesity in community-dwelling elderly women: the EPIDOS (EPIDemiologie de l'OSteoporose) Study. Am J Clin Nutr.

[38] Guralnik JM, LaCroix AZ, Branch LG, Kasl SV, Wallace RB (1991). Morbidity and disability in older persons in the years prior to death. Am J Public Health.

[39] Hirvensalo M, Rantanen T, Heikkinen E (2000). Mobility difficulties and physical activity as predictors of mortality and loss of independence in the community-living older population. J Am Geriatr Soc.

[40] Batsis JA, Zbehlik AJ, Scherer EA, Barre LK, Bartels SJ (2015). Normal weight with central obesity, physical activity, and functional decline: data from the osteoarthritis initiative. J Am Geriatr Soc.

[41] Ferrucci L, Penninx BW, Volpato S, Harris TB, Bandeen-Roche K, Balfour J, Leveille SG, Fried LP, Md JM (2002). Change in muscle strength explains accelerated decline of physical function in older women with high interleukin-6 serum levels. J Am Geriatr Soc.

[42] Holstege MS, Lindeboom R, Lucas C (2011). Preoperative quadriceps strength as a predictor for short-term functional outcome after total hip replacement. Arch Phys Med Rehabil.

[43] Park SW, Goodpaster BH, Strotmeyer ES, de Rekeneire N, Harris TB, Schwartz AV, Tylavsky FA, Newman AB (2006). Decreased muscle strength and quality in older adults with type 2 diabetes: the health, aging, and body composition study. Diabetes.

[44] Al Snih S, Markides KS, Ottenbacher KJ, Raji MA (2004). Hand grip strength and incident ADL disability in elderly Mexican Americans over a seven-year period. Aging Clin Exp Res.

[45] Stenholm S, Harkanen T, Sainio P, Heliovaara M, Koskinen S (2012). Long-term changes in handgrip strength in men and women--accounting the effect of right censoring due to death. J Gerontol A Biol Sci Med Sci.

[46] Martien S, Delecluse C, Boen F, Seghers J, Pelssers J, Van Hoecke AS, Van Roie E (2014). Is knee extension strength a better predictor of functional performance than handgrip strength among older adults in three different settings?. Arch Gerontol Geriatr.

[47] Batsis JA, Barre LK, Mackenzie TA, Pratt SI, Lopez-Jimenez F, Bartels SJ (2013). Variation in the prevalence of sarcopenia and sarcopenic obesity in older adults associated with different research definitions: dual-energy X-ray absorptiometry data from the National Health and Nutrition Examination Survey 1999-2004. J Am Geriatr Soc.

[48] Romero-Corral A, Somers VK, Sierra-Johnson J, Thomas RJ, Collazo-Clavell ML, Korinek J, Allison TG, Batsis JA, Sert-Kuniyoshi FH, Lopez-Jimenez F (2008). Accuracy of body mass index in diagnosing obesity in the adult general population. Int J Obes (Lond).

