# Supplementary material for: Dynapenic obesity and the effect on long-term physical function and quality of life: data from the osteoarthritis initiative
Source: BMC Geriatr. 2015 Oct 8;15:118. doi: 10.1186/s12877-015-0118-9 (PMC4599326; doi:10.1186/s12877-015-0118-9)
Supplement: Additional file 1: — Sensitivity analysis of excluded vs. Included participants. (DOCX 15 kb) [file 12877_2015_118_MOESM1_ESM.docx]

**Additional file 1: Sensitivity Analysis of Excluded vs. Included Participants**

|  | Excluded | Included | p-value |
| --- | --- | --- | --- |
|  | N=560 | N=2025 |  |
| **Age, years** | 69.1 ± 5.5 | 68.2±5.4 | <0.001 |
| **Female sex** | 322 (57.5) | 1,269 (62.7) | 0.03 |
| **Education Status** |  |  |  |
| **< High School** | 129 (23.2) | 384 (19.0) |  |
| **Some College** | 136 (24.5) | 483 (23.9) |  |
| **College** | 97 (17.5 ) | 395(19.6) | 0.13 |
| **>College** | 194 (34.9) | 756 (37.5) |  |
| **Yearly Income** |  |  |  |
| **>$50,000** | 247 (47.3) | 1,046 (54.4) | 0.004 |
| **Marital Status** |  |  |  |
| **Married** | 371 (66.7) | 1,340 (66.4) | 0.88 |
| **Race** |  |  |  |
| **White** | 425 (76.2) | 1,710 (84.4) |  |
| **Black** | 115 (20.6) | 268 (13.2) |  |
| **other** | 18 (3.2) | 47 (2.3) | <0.001 |
| **Charlson Score** | 0.62 ± 1.0 | 0.43 ±0.88 | <0.001 |
| **Baseline WOMAC Rt** | 15.6±15.9 | 11.0±13.6 | <0.001 |
| **Baseline WOMAC Left** | 16.0±17.4 | 10.6±14.4 | <0.001 |
| **Ever Smoker** | 297 (22.9) | 999 (77.1) | 0.13 |
| **Number of Medications** | 4.25±2.7 | 3.80±2.46 | <0.001 |
| **Body mass index, kg/m^2^** |  |  |  |
| **Gait speed, m/s** | 1.24±0.21 | 1.29±0.21 | <0.001 |
| **PASE baseline** | 133.5±69.8 | 138.1 ± 67.3 | 0.16 |
| **SF-12 PCS** | 45.6±10.0 | 49.0±8.7 | <0.001 |
| **SF-12 MCS** | 55.3±7.4 | 54.8±7.5 | 0.15 |
| **400M walk test, seconds** | 396.4±28.5 | 397.1±26.5 | 0.58 |
|  |  |  |  |

All values represent mean ± SD, or count (%)

P-value represents the difference between the excluded and included analytical cohort

MCS – Mental Component Score; PASE - Physical Activity Scale for the Elderly; PCS – Physical Component Score; SF – Short Form ; WOMAC – Western Ontario and McMaster University Arthritis Index
