# Supplementary material for: Dynapenic obesity and the effect on long-term physical function and quality of life: data from the osteoarthritis initiative
Source: BMC Geriatr. 2015 Oct 8;15:118. doi: 10.1186/s12877-015-0118-9 (PMC4599326; doi:10.1186/s12877-015-0118-9)
Supplement: Additional file 2: — a: Age-Stratified Functional Outcome - Males. b: Age-Stratified Functional Outcome - Females. (DOCX 34 kb) [file 12877_2015_118_MOESM2_ESM.docx]

**Additional file 2**

**a: Age-Stratified Functional Outcome - Males**

|  | **Gait Speed** | | | | **400M Walk** | | | **LLFDI-Frequency** | | | **LLFDI-Limitation** | | | **SF-12 PCS** | | | | **SF-12 MCS** | | |
| --- | --- | --- | --- | --- | --- | --- | --- | --- | --- | --- | --- | --- | --- | --- | --- | --- | --- | --- | --- | --- |
| **60-70years** | **β** | **SE** | **p** | **β** | | **SE** | **p** | **β** | **SE** | **p** | **β** | **SE** | **p-value** | **β** | **SE** | **p** | **β** | | **SE** | **p** |
|  |  |  |  |  | |  |  |  |  |  |  |  |  |  |  |  |  | |  |  |
| **Intercept** | 1.27 | 0.06 | <0.001 | 401.4 | | 19.9 | <0.001 | 49.8 | 1.06 | <0.001 | 75.3 | 2.79 | <0.001 | 43.0 | 7.93 | <0.001 | 45.0 | | 7.9 | <0.001 |
| **Dyn Obes** | -0.13 | 0.03 | <0.001 | 2.28 | | 2.93 | 0.44 | 0.98 | 0.55 | 0.08 | 0.15 | 1.46 | 0.92 | -4.41 | 1.18 | <0.001 | 0.60 | | 1.16 | 0.60 |
| **Dyn No Obes** | -0.05 | 0.2 | 0.04 | 0.68 | | 2.24 | 0.76 | -1.16 | 0.39 | 0.003 | -0.98 | 1.02 | 0.33 | -1.05 | 0.89 | 0.24 | -0.18 | | 0.89 | 0.84 |
| **Obes No Dyn** | -0.08 | 0.02 | <0.001 | 2.13 | | 1.89 | 0.26 | -0.39 | 0.32 | 0.23 | -3.03 | 0.85 | <0.001 | -2.23 | 0.75 | 0.003 | -0.30 | | 0.74 | 0.68 |
| **No Dyn No Obes** | Ref | ref | Ref | Ref | | ref | Ref | Ref | Ref | ref | ref | ref | ref | Ref | Ref | Ref | ref | | ref | ref |
|  |  |  |  |  | |  |  |  |  |  |  |  |  |  |  |  |  | |  |  |
| **Time** | -0.006 | 0.0023 | 0.008 | 0.27 | | 0.18 | 0.13 | --- | --- | --- | --- | --- | --- | -0.26 | 0.13 | 0.05 | -0.09 | | 0.13 | 0.47 |
| **x Dyn Obes** | 0.0009 | 0.006 | 0.88 | -0.27 | | 0.45 | 0.54 | --- | --- | --- | --- | --- | --- | -0.05 | 0.34 | 0.87 | -0.14 | | 0.32 | 0.66 |
| **X Dyn No Obes** | 0.006 | 0.004 | 0.15 | -1.04 | | 0.34 | 0.002 | --- | --- | --- | --- | --- | --- | -0.04 | 0.26 | 0.88 | 0.57 | | 0.24 | 0.02 |
| **x Obes No Dyn** | 0.003 | 0.004 | 0.37 | -0.27 | | 0.29 | 0.35 | --- | --- | --- | --- | --- | --- | -0.09 | 0.22 | 0.67 | -0.17 | | 0.20 | 0.41 |
| **x No Dyn No Obes** | Ref | Ref | ref | Ref | | Ref | ref | --- | --- | --- | --- | --- | --- | ref | ref | ref | ref | | ref | ref |
|  |  |  |  |  | |  |  |  |  |  |  |  |  |  |  |  |  | |  |  |
|  |  |  |  |  | |  |  |  |  |  |  |  |  |  |  |  |  | |  |  |
| **≥70 years old** | **β** | **SE** | **p** | **β** | | **SE** | **p** | **β** | **SE** | **p** | **β** | **SE** | **p** | **β** | **SE** | **p** | **β** | | **SE** | **p** |
| **Intercept** | 1.25 | 0.14 | <0.001 | 348.4 | | 41.6 | <0.001 | 35.2 | 2.66 | <0.001 | 78.1 | 7.17 | <0.001 | 63.9 | 11.7 | <0.001 | 74.6 | | 9.2 | <0.001 |
| **Dyn Obes** | -0.16 | 0.04 | <0.001 | -23.8 | | 4.9 | <0.001 | 2.73 | 0.64 | <0.001 | -12.7 | 1.72 | <0.001 | -7.65 | 1.36 | <0.001 | -0.79 | | 1.07 | 0.46 |
| **Dyn No Obes** | -0.05 | 0.02 | 0.02 | 0.44 | | 2.98 | 0.88 | -0.38 | 0.35 | 0.29 | -5.73 | 0.96 | <0.001 | -1.21 | 0.86 | 0.16 | -0.37 | | 0.68 | 0.58 |
| **Obes No Dyn** | -0.08 | 0.03 | 0.004 | -4.73 | | 3.50 | 0.18 | 0.10 | 0.41 | 0.81 | -6.11 | 1.11 | <0.001 | -2.65 | 1.02 | 0.009 | -2.06 | | 0.80 | 0.01 |
| **No Dyn No Obes** | ref | Ref | ref | ref | | Ref | ref | Ref | Ref | ref | Ref | Ref | ref | Ref | Ref | ref | ref | | ref | ref |
|  |  |  |  |  | |  |  |  |  |  |  |  |  |  |  |  |  | |  |  |
| **Time** | -0.014 | 0.003 | <0.001 | -0.81 | | 0.89 | 0.36 | --- | --- | --- | --- | --- | --- | -0.65 | 0.14 | <0.001 | -0.10 | | 0.13 | 0.46 |
| **x Dyn Obes** | -0.003 | 0.006 | 0.63 | -17.2 | | 2.19 | <0.001 | --- | --- | --- | --- | --- | --- | 0.001 | 0.34 | 0.99 | 0.20 | | 0.31 | 0.52 |
| **X Dyn No Obes** | -0.002 | 0.0047 | 0.52 | -0.29 | | 1.32 | 0.83 | --- | --- | --- | --- | --- | --- | -0.042 | 0.22 | 0.06 | -0.06 | | 0.20 | 0.75 |
| **x Obes No Dyn** | 0.0005 | 0.004 | 0.91 | -1.45 | | 1.55 | 0.35 | --- | --- | --- | --- | --- | --- | -0.08 | 0.26 | 0.76 | 0.06 | | 0.23 | 0.79 |
| **x No Dyn No Obes** | Ref | Ref | ref | Ref | | ref | ref | --- | --- | --- | --- | --- | --- | ref | ref | ref | ref | | ref | ref |
|  |  |  |  |  | |  |  |  |  |  |  |  |  |  |  |  |  | |  |  |

All linear mixed models are adjusted for physical activity (Physical Activity Scale for the Elderly Score), smoking status, Charlson co-morbidity score, education, race, cohort type (incidence, progression, control). Referent category is the neither dynapenia nor obesity group. Time-dependent co-variates are included in time x group interaction. LLDI was only available at 4-year follow-up thereby no time interaction term model was considered for this outcome measure.

Abbreviations: Dyn: Dynapenia; Obes: Obesity

**β** – beta-coefficient of regression model; SE– Standard errors; LLDI – Late-Life Functional and Disability Index; MCS – Mental Component Score; PCS – Physical Component Score; SF – Short Form

**b: Age-Stratified Functional Outcome - Females**

|  | **Gait Speed** | | | **400M Walk** | | | **LLFDI-Frequency** | | | **LLFDI-Limitation** | | | **SF- 12 PCS** | | | **SF-12 MCS** | | |
| --- | --- | --- | --- | --- | --- | --- | --- | --- | --- | --- | --- | --- | --- | --- | --- | --- | --- | --- |
| **60-70years** | **β** | **SE** | **p** | **β** | **SE** | **p** | **β** | **SE** | **p** | **β** | **SE** | **p** | **β** | **SE** | **p** | **β** | **SE** | **p** |
| **Intercept** | 1.21 | 0.06 | <0.001 | 385.3 | 21.54 | <0.001 | 50.1 | 1.14 | <0.001 | 60.4 | 2.6 | <0.001 | 36.7 | 6.4 | <0.001 | 34.5 | 6.4 | <0.001 |
| **Dyn Obes** | -0.15 | 0.023 | <0.001 | -2.55 | 3.40 | 0.45 | -0.46 | 0.45 | 0.31 | -5.67 | 1.04 | <0.001 | -5.90 | 1.00 | <0.001 | -1.09 | 0.99 | 0.27 |
| **Dyn No Obes** | -0.045 | 0.16 | 0.005 | 1.30 | 2.32 | 0.58 | -0.03 | 0.30 | 0.92 | -3.75 | 0.69 | <0.001 | -2.94 | 0.69 | <0.001 | -0.37 | 0.68 | 0.59 |
| **Obes No Dyn** | -0.08 | 0.015 | <0.001 | -1.81 | 2.12 | 0.39 | -0.73 | 0.28 | 0.01 | -4.46 | 0.64 | <0.001 | -2.19 | 0.64 | 0.001 | -0.72 | 0.63 | 0.25 |
| **No Dyn No Obes** | Ref | ref | ref | Ref | Ref | Ref |  |  |  | Ref | Ref | ref | Ref | rEf | ref | ref | ref | ref |
|  |  |  |  |  |  |  |  |  |  |  |  |  |  |  |  |  |  |  |
| **Time** | -0.0041 | 0.002 | 0.023 | -0.631 | 0.37 | 0.09 | --- | --- | --- | --- | --- | --- | -0.13 | 0.10 | 0.19 | 0.003 | 0.10 | 0.98 |
| **x Dyn Obes** | -0.00026 | 0.0045 | 0.95 | 0.30 | 0.98 | 0.76 | --- | --- | --- | --- | --- | --- | -0.03 | 0.24 | 0.89 | 0.30 | 0.25 | 0.23 |
| **X Dyn No Obes** | -0.0075 | 0.0032 | 0.02 | 0.14 | 0.68 | 0.83 | --- | --- | --- | --- | --- | --- | -0.18 | 0.17 | 0.30 | -0.17 | 0.18 | 0.33 |
| **x Obes No Dyn** | -0.00001 | 0.003 | 0.99 | -0.758 | 0.59 | 0.20 | --- | --- | --- | --- | --- | --- | -0.42 | 0.15 | 0.006 | 0.02 | 0.16 | 0.92 |
| **x No Dyn No Obes** | Ref | Ref | ref | Ref | Ref | ref | --- | --- | --- | --- | --- | --- | ref | ref | ref | ref | ref | Ref |
|  |  |  |  |  |  |  |  |  |  |  |  |  |  |  |  |  |  |  |
| **≥70 years old** | **β** | **SE** | **p** | **β** | **SE** | **p** | **β** | **SE** | **p** | **β** | **SE** | **p** | **β** | **SE** | **p** | **β** | **SE** | **p** |
| **Intercept** | 1.06 | 0.08 | <0.001 | 557.0 | 56.3 | <0.001 | 60.4 | 1.31 | <0.001 | 79.0 | 3.24 | <0.001 | 60.5 | 9.9 | <0.001 | 44.0 | 8.8 | <0.001 |
| **Dyn Obes** | -0.123 | 0.032 | <0.001 | -11.0 | 7.7 | 0.15 | -0.95 | 0.52 | 0.07 | -0.49 | 1.28 | 0.70 | -3.22 | 1.32 | 0.01 | 0.38 | 1.17 | 0.74 |
| **Dyn No Obes** | -0.033 | 0.02 | 0.10 | 3.11 | 4.61 | 0.50 | -0.31 | 0.32 | 0.33 | -0.01 | 0.79 | 0.99 | 0.25 | 0.82 | 0.76 | -0.44 | 0.73 | 0.55 |
| **Obes No Dyn** | -0.09 | 0.03 | 0.001 | -13.4 | 6.12 | 0.03 | -1.36 | 0.42 | 0.001 | -3.41 | 1.03 | 0.001 | -3.69 | 1.07 | 0.001 | 0.71 | 0.95 | 0.46 |
| **No Dyn No Obes** | Ref | ref | ref | Ref | Ref | ref | ref | ref | ref | ref | Ref | Ref | ref | ref | Ref | ref | ref | ref |
|  |  |  |  |  |  |  |  |  |  |  |  |  |  |  |  |  |  |  |
| **Time** | -0.015 | 0.002 | <0.001 | -2.34 | 1.06 | 0.03 | --- | --- | --- | --- | --- | --- | -0.79 | 0.12 | <0.001 | -0.29 | 0.12 | 0.02 |
| **x Dyn Obes** | -0.0097 | 0.006 | 0.08 | -6.08 | 2.93 | 0.038 | --- | --- | --- | --- | --- | --- | 0.26 | 0.30 | 0.39 | 0.15 | 0.30 | 0.60 |
| **X Dyn No Obes** | 0.00087 | 0.0035 | 0.8 | 0.64 | 1.64 | 0.69 | --- | --- | --- | --- | --- | --- | 0.24 | 0.19 | 0.20 | 0.07 | 0.19 | 0.72 |
| **x Obes No Dyn** | -0.0043 | 0.0045 | 0.34 | -5.02 | 2.18 | 0.02 | --- | --- | --- | --- | --- | --- | -0.41 | 0.25 | 0.09 | 0.31 | 0.24 | 0.21 |
| **x No Dyn No Obes** | Ref | Ref | ref | Ref | ref | ref | --- | --- | --- | --- | --- | --- | ref | ref | ref | ref | ref | ref |

All linear mixed models are adjusted for physical activity (Physical Activity Scale for the Elderly Score), smoking status, Charlson co-morbidity score, education, race, cohort type (incidence, progression, control). Referent category is the neither dynapenia nor obesity group. Time-dependent co-variates are included in time x group interaction. LLDI was only available at 4-year follow-up thereby no time interaction term model was considered for this outcome measure.

Abbreviations: Dyn - Dynapenia; Obes: Obesity

**β** – beta-coefficient of regression model; SE– Standard errors; LLDI – Late-Life Functional and Disability Index; MCS – Mental Component Score; PCS – Physical Component Score; SF – Short Form
